# Role of Maturation of Lipoproteins in the Pathogenesis of the Infection Caused by *Streptococcus suis* Serotype 2

**DOI:** 10.3390/microorganisms9112386

**Published:** 2021-11-19

**Authors:** Servane Payen, David Roy, Anaïs Boa, Masatoshi Okura, Jean-Philippe Auger, Mariela Segura, Marcelo Gottschalk

**Affiliations:** 1 Swine and Poultry Infectious Diseases Research Center (CRIPA) and Research Group on Infectious Diseases in Production Animals (GREMIP), Faculty of Veterinary Medicine, University of Montreal, Saint-Hyacinthe, QC J2S 2M2, Canada; servane.payen@umontreal.ca (S.P.); dav.roy@gmail.com (D.R.); anais.boa@umontreal.ca (A.B.); jean-philippe.auger.1@umontreal.ca (J.-P.A.); mariela.segura@umontreal.ca (M.S.); 2 Division of Bacterial and Parasitic Disease, National Institute of Animal Health, National Agriculture and Food Research Organization, Tsukuba 305-0856, Japan; mokura@affrc.go.jp

**Keywords:** *Streptococcus suis*, lipoproteins, maturation, inflammation

## Abstract

*Streptococcus suis* serotype 2 is an important porcine bacterial pathogen associated with multiple pathologies in piglets. Bacterial lipoproteins (LPPs) have been described as playing important roles in the pathogenesis of the infection of other Gram-positive bacteria as adhesins, pro-inflammatory cell activators and/or virulence factors. In the current study, we aimed to evaluate the role of the prolipoprotein diacylglyceryl transferase (Lgt) and lipoprotein signal peptidase (Lsp) enzymes, which are responsible for LPP maturation, on the pathogenesis of the infection caused by two different sequence types (STs) of *S. suis* serotype 2 strains (virulent ST1 and highly virulent ST7). Through the use of isogenic Δ*lgt*, Δ*lsp* and double Δ*lgt*/Δ*lsp* mutants, it was shown that lack of these enzymes did not influence *S. suis* adhesion/invasion to porcine respiratory epithelial cells. However, in the absence of the Lsp and/or Lgt, a significant reduction in the capacity of *S. suis* to activate phagocytic cells and induce pro-inflammatory mediators (in vitro and in vivo) was observed. In general, results obtained with the double mutant did not differ in comparison to single mutants, indicating lack of an additive effect. Finally, our data suggest that these enzymes play a differential role in virulence, depending on the genetic background of the strain and being more important for the highly virulent ST7 strain.

## 1. Introduction

*Streptococcus suis* is one of the most important swine pathogens, causing meningitis, endocarditis, arthritis or septicemia with sudden death in weaned piglets. Additionally, it is an emerging zoonotic agent causing disease in humans with clinical manifestations similar to those observed in piglets [1]. Presently, 29 serotypes have been described, with serotype 2 being the most commonly isolated from humans and diseased animals [2]. However, serotype 2 strains are heterogeneous and belong to different sequence types (STs), as determined by multi-locus sequence typing [3]. ST1 strains are virulent and are mostly associated with disease in both pigs and humans in Europe and Asia. In comparison, ST7 strains are highly virulent and responsible for human outbreaks in China, with fatality rates nearing 20% [4]. Of the various virulence factors described for *S. suis* serotype 2, the capsular polysaccharide (CPS) is considered an essential but insufficient critical virulence factor [5]. Non-encapsulated mutants are avirulent, but natural low virulent field strains are still well encapsulated [6]. Suilysin, the most important toxin produced by *S. suis*, plays different roles during the pathogenesis of the infection but is not a critical virulence factor, at least for infection in pigs [7]. An arsenal of surface proteins has been proposed as being important for virulence, although most of these have not been confirmed [5].

Although knowledge on the pathogenesis of the infection caused by *S. suis* has improved in recent years, it remains incomplete [1]. It is well known that inflammation plays a critical role in the development of *S. suis* diseases [1]. The *S. suis* CPS is, by itself, a poor activator of the innate immune system [8]. Therefore, the main components of *S. suis* involved in cell activation are present in the bacterial cell wall [9]. In the past, lipoteichoic acid (LTA) was suggested to be one of the main *S. suis* activators of the innate immune system [10]. However, a recent study showed that the co-purified lipoproteins (LPPs), rather than LTA, are the main activators following recognition by the Toll-like receptor 2 (TLR2), which is also the case in many other Gram-positive pathogens [11,12,13,14].

In addition, LPPs often play important roles in pathogen/host interactions during the development of the infection [9,15]. Bacterial LPPs belong to an important family of proteins that are anchored to cell membranes, where they carry out diverse functions [12,16]. Bacterial LPPs share a structural similarity due to a unique maturation process that is preserved in all species [17]. LPPs of Gram-positive bacteria are processed by two key enzymes: prolipoprotein diacylglyceryl transferase (Lgt) and lipoprotein signal peptidase (Lsp). The Lgt enzyme recognizes a so-called lipobox motif (LXXC) in the C-terminal region of the signal peptide of a premature lipoprotein and transfers a diacylglyceryl moiety to the cysteine residue of the lipobox [17]. Subsequently, the Lsp enzyme cleaves the signal peptide resulting in a mature lipoprotein. Mature LPPs are mainly found attached to the periplasmic side of the plasma membrane [16]. Nevertheless, recent studies have shown that certain LPPs are either passively released into the extracellular medium or are actively secreted by a mechanism that is still poorly understood [14,18]. In pathogenic streptococci, structural changes brought about by the maturation process have been suggested to modify the interactions with host cells [19,20,21] with variable effects on virulence [14,22,23]. However, little information is available regarding the role of LPP maturation in the pathogenesis of the infection caused by *S. suis* [24,25]. In the current study, we evaluated the role of the Lgt and Lsp enzymes in *S. suis* adhesion/invasion of epithelial cells, bacterial survival in blood, in vitro and in vivo induction of inflammatory mediators and virulence, using virulent (ST1) and highly virulent (ST7) serotype 2 strains.

## 2. Materials and Methods

### 2.1. Ethics Statement

This study was carried out in accordance with the recommendations of the guidelines and policies of the Canadian Council on Animal Care and the principles set forth in the Guide for the Care and Use of Laboratory Animals. The protocols and procedures were approved by the Animal Welfare Committee of the University of Montreal (permit number Rech-1570).

### 2.2. Bacterial Strains and Growth Conditions

The wild-type *S. suis* serotype 2 virulent P1/7 (ST1) and highly virulent SC84 (ST7) strains, previously used in our studies [26], and their lipoprotein maturation isogenic mutants (see below) used in this study are listed in Table 1. *S. suis* strains were cultured in Todd Hewitt broth (THB; Becton Dickinson, Mississauga, ON, Canada) as previously described [27]. The *Escherichia coli* strains, and different plasmids used in this study are also listed in Table 1. For in vitro cell culture assays, bacteria were prepared as previously described [10,28] and resuspended in cell culture medium. When needed, antibiotics (Sigma-Aldrich, Oakville, ON, Canada) were added to the media at the following concentrations: for *S. suis*, spectinomycin (Spc) at 100 μg/mL; for *E. coli*, kanamycin (Km) and spectinomycin at 50 μg/mL and ampicillin (Ap) at 100 μg/mL. For experimental infections, early stationary phase bacteria were washed twice in phosphate-buffered saline, pH 7.4, and resuspended in THB [29,30,31]. Bacterial cultures were appropriately diluted and plated on THB agar (THA) to accurately determine bacterial concentrations.

### 2.3. DNA Manipulations

Genomic DNA was extracted from the *S. suis* wild-type strains using InstaGene Matrix solution (BioRad Laboratories, Hercules, CA, USA). Preparations of plasmid DNA were carried out using the QIAprep Spin Miniprep Kit (Qiagen, Valencia, CA, USA). Restriction enzymes and DNA-modifying enzymes (Fisher Scientific, Ottawa, ON, Canada) were used according to the manufacturer’s recommendations. Oligonucleotide primers (Table 2) were obtained from Integrated DNA Technologies (Coralville, IA, USA) and PCRs were carried out with the iProof proofreading DNA polymerase (BioRad Laboratories, Mississauga, ON, Canada) or the Taq DNA polymerase (Qiagen). Amplification products were purified using the QIAquick PCR Purification Kit (Qiagen) and sequenced using an ABI 310 Automated DNA Sequencer and ABI PRISM Dye Terminator Cycle Sequencing Kit (Applied Biosystems, Carlsbad, CA, USA).

### 2.4. Construction of the Lipoprotein Maturation Isogenic Mutants

Precise in-frame deletion of the *lgt* and *lsp* genes from strains P1/7 and SC84 were constructed using splicing-by-overlap-extension PCRs as previously described [37,38]. Overlapping PCR products were cloned into pCR2.1 (Invitrogen, Burlington, ON, Canada), extracted with EcoRI, recloned into the thermosensitive *E. coli*–*S. suis* shuttle plasmid pSET4s, and digested with the same enzyme, giving rise to the knockout vector p4Δ*lgt* or p4Δ*lps*. Electroporation of these vectors into wild-type strains P1/7 and SC84 and procedures for construction of the mutants were previously described [35]. Allelic replacement was confirmed by PCR and DNA sequencing analyses. Amplification products were purified with the QIAgen PCR Purification Kit (Qiagen) and sequenced as described above.

### 2.5. Complementation of the Mutant Strains

The pMX1 vector was used for the generation of recombinant plasmids for complementation analyses (Table 1). This vector is a derivative of the *E. coli-S. suis* shuttle cloning vector pSET2 [39] and possesses the *S. suis malX* promoter for transgene expression in *S. suis*. The entire *lgt* and *lsp* genes were amplified from genomic DNA of the *S. suis* P1/7 and SC84 strains and cloned into pMX1 via EcoRI and NcoI sites, generating complementation vectors pMX1-*lgt*, and pMX1-*lsp*. These plasmids were introduced into *E. coli* MC1061 for verification of the sequences and then into the respective deletion mutants derived from *S. suis* P1/7 and SC84 to construct the *lgt*- and *lsp*-complemented mutants.

### 2.6. Growth Analysis

Overnight cultures of wild-type and mutant strains were diluted in fresh THB or plasma and growth was followed during 24 h of incubation at 37 °C. The total number of CFU/mL was evaluated at different incubation times.

### 2.7. Bacterial Surface Hydrophobicity Assay

Relative surface hydrophobicity of the *S. suis* wild-type strains and non-encapsulated mutants was determined by measuring adsorption to *n*-hexadecane as previously described [40].

### 2.8. Preparation of Heat-Killed S. suis

Heat-killed *S. suis* suspensions were prepared as previously described [41]. Briefly, *S. suis* was cultured to mid-log phase and then incubated at 60 °C for 45 min. A lack of viability was confirmed by culturing on blood agar plates at 37 °C for 48 h. Heat-killed *S. suis* were resuspended in cell culture medium at a concentration equivalent to 2 × 10^9^ CFU/mL prior to bone marrow dendritic cell stimulation.

### 2.9. Preparation of Bacterial Supernatants

Bacteria were grown to mid-log phase (OD_600nm_ = 0.6), and growth was immediately stopped on ice. Bacterial cultures were appropriately diluted and plated on THB agar (THA) to accurately determine bacterial concentrations. Bacteria were then centrifuged for 15 min at 3312× *g* at 4 °C to separate bacteria from the medium. Supernatants were collected and filtered using 0.2 µm filters. Supernatants were then applied to an Amicon^®^ Ultra-15 10K centrifugal filter and resuspended in cell culture medium prior to bone-marrow dendritic cell stimulation. Absence of bacteria in supernatants was confirmed by culturing on blood agar plates at 37 °C for 48 h.

### 2.10. Porcine Tracheal Epithelial Cell Culture and Bacterial Adhesion and Invasion Assays

The neonatal porcine tracheal epithelial cell line (NPTr), frequently used in *S. suis* studies, was used and cultured until confluence as previously described [42]. Cells were infected with 1 × 10^6^ CFU/well (multiplicity of infection (MOI) = 10) of the different *S. suis* strains and incubated for 2 or 4 h at 37 °C in 5% CO_2_. The adhesion assay, which quantifies total cell-associated bacteria (surface-adherent and intracellular bacteria), and invasion assay (using the antibiotic protection assay) were performed as previously described [42].

### 2.11. Whole Blood Bactericidal (Killing) Assay

Blood was collected from six- to ten-week-old C57BL/6J (Jackson Research Laboratories, Bar Harbor, ME, USA) mice and mixed with sodium heparin (Sigma-Aldrich). The test was performed as previously described [29], with a few modifications. Briefly, leukocytes (9 × 10^6^ cells/mL on average) were transferred to a microtube containing around 1 × 10^7^ CFU/mL of the different *S. suis* strains (MOI = 1) and incubated for 2 h, mixing every 20 min. After incubation, cells were lysed, and appropriate dilutions plated on THA to determine viable bacterial counts. Resistance to bacterial killing by blood leukocytes was compared to incubation in plasma alone (obtained by centrifuging whole blood at 1800× *g* for 10 min at 4 °C). The percentage of bacteria killed was determined using the following formula: 1 − (bacteria in blood/bacteria in plasma)/100%.

### 2.12. Generation of Bone Marrow-Derived Dendritic Cells (bmDC)

The femur and tibia from C57BL/6J mice (Jackson) were used to generate bmDCs as previously described [10]. Briefly, hematopoietic bone marrow stem cells were cultured in complete culture medium (RPMI-1640 supplemented with 5% heat-inactivated fetal bovine serum, 10 mM HEPES, 2 mM L-glutamine and 50 µM 2-mercaptoethanol (Gibco, Burlington, ON, Canada)) complemented with 20% granulocyte-macrophage colony-stimulating factor from mouse-transfected Ag8653 cells [43]. Cell purity was confirmed to be at least 90% CD11c+ by flow cytometry as previously described [10]. Albeit this culture system cannot completely rule out the presence of other innate cells such as macrophages, it represents an enriched source of bmDCs [44].

### 2.13. S. suis Infection of bmDCs

All experiments were performed in the absence of endotoxin (lipopolysaccharide) contamination and under non-toxic conditions, the latter being evaluated by lactate dehydrogenase release with the CytoTox 96^®^ Non-Radioactive Cytotoxicity Assay (Promega, Madison, WI, USA). Prior to infection, cells were resuspended at 1 × 10^6^ cells/mL in complete medium and stimulated with the different live *S. suis* strains (1 × 10^6^ CFU/mL; initial MOI = 1). Conditions used were based on those previously published [10,27]. A higher MOI (corresponding to 100) was used with heat-killed bacteria. Supernatants were collected at 4, 6, 8 and 12 h following infection with *S. suis* (live, heat-killed or bacterial-free supernatant), incubation times at which secreted cytokine levels were maximal in the absence of cytotoxicity as determined by lactate dehydrogenase release (data not shown) [10,27]. Mock-infected cells served as negative controls. Secreted levels of tumor necrosis factor (TNF), interleukin (IL)-6, C-C motif chemokine ligand (CCL) 3 and C-X-C motif chemokine ligand (CXCL) 1 were quantified by sandwich ELISA using pair-matched antibodies from R&D Systems (Minneapolis, MN, USA) according to the manufacturer’s recommendations.

### 2.14. S. suis Virulence Mouse Model of Systemic Infection

A C57BL/6J mouse model of infection was used. These studies were carried out in strict accordance with the recommendations of and approved by the University of Montreal Animal Welfare Committee guidelines and policies, including euthanasia to minimize animal suffering through the use of humane endpoints, applied throughout this study when animals were seriously affected since mortality was not an endpoint measurement. Sixty 6-week-old female C57BL/6J (Jackson) were used for these experiments (15 mice per group). Mice were inoculated with 1 × 10^7^ CFU via the intraperitoneal route and health and behavior monitored at least thrice daily until 72 h post-infection and twice thereafter until the end of the experiment (12 days post-infection) for the development of clinical signs of sepsis, such as depression, swollen eyes, rough hair coat, prostration and lethargy. For bacteremia studies, blood samples were collected from the caudal vein of surviving mice 12, 24 and 48 h post-infection and plated as previously described [29].

### 2.15. Measurement of Plasma (Systemic) Pro-Inflammatory Mediators

In parallel (another experiment), eight mice per group were intraperitoneally infected with 1 × 10^7^ CFU and blood was collected 12 h post-infection (p.i.) by intracardiac puncture following euthanasia and anti-coagulated with EDTA (Sigma-Aldrich) as previously described [29,45]. Plasma supernatants were collected following centrifugation at 10,000× *g* for 10 min at 4 °C and stored at −80 °C. The 12 h post-infection time point was selected to obtain maximal pro-inflammatory mediator production in the absence of significant mouse mortality as previously shown [29]. Plasmatic concentrations of IL-6, IL-12p70, G-CSF, interferon (IFN)-γ, CCL2, CCL3, CCL4, CCL5, CXCL2 and CXCL9 were measured using a custom-made cytokine Bio-Plex Pro™ assay (Bio-Rad, Hercules, CA, USA) according to the manufacturer’s instructions. Acquisition was performed on the MAGPIX platform (Luminex^®^) and data analyzed using the Bio-Plex Manager 6.1 software (Bio-Rad).

### 2.16. Statistical Analyses

Normality of data was verified using the Shapiro–Wilk test. Accordingly, parametric (unpaired *t* test) or non-parametric tests (Mann–Whitney rank sum test), where appropriate, were performed to evaluate statistical differences between groups. Log-rank test was used to compare survival rates between wild-type-infected mice and those infected with mutant strains. Each in vitro test was repeated in at least three independent experiments. *p* < 0.05 was considered as statistically significant.

## 3. Results

### 3.1. Characteristics of the Δlgt, Δlsp and Δlgt/Δlsp Mutants: Normal Growth in Rich Medium and in Plasma

**Figure 1:**
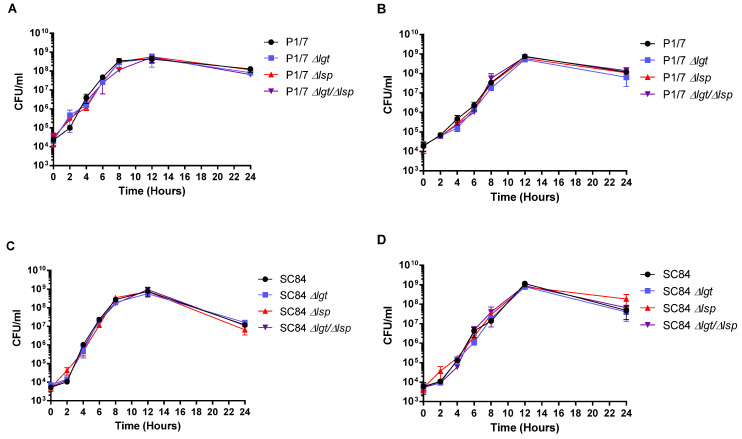
Growth of the *S. suis* serotype 2 wild-type virulent strain P1/7 (ST1) and highly virulent strain SC84 (ST7) (black) in THB (**A**,**C**) and plasma (**B**,**D**) as well as their respective Δ*lgt* (blue), Δ*lsp* (red) and Δ*lgt*/Δ*lsp* (purple) mutants. Each point represents mean bacterial concentration (CFU/mL) +/− SEM (*n* = 3 independent experiments).

The Δ*lgt* and Δ*lsp* mutants, as with the Δ*lgt*/Δ*lsp* double mutant, presented general characteristics similar to those of their respective wild-type strains. All mutants remained well encapsulated as shown by the presence of hydrophobicity values between 5.5% and 9.3%, similar to those of wild–type strains P1/7 and SC84 (4.3% and 6.7%, respectively). In addition, all mutants remained typable (serotype 2) by the co-agglutination test [46]. Finally, bacterial growth in both rich medium and plasma was evaluated. Although growth in plasma presented a certain delay for all strains during the exponential phase (when compared to growth in rich medium), no significant differences were observed between the mutants and their respective wild-type strains in both conditions (Figure 1A–D).

### 3.2. Absence of the Lipoprotein Maturation Enzymes Does Not Affect S. suis Serotype 2 Adhesion to and Invasion of Respiratory Epithelial Cells

**Figure 2:**
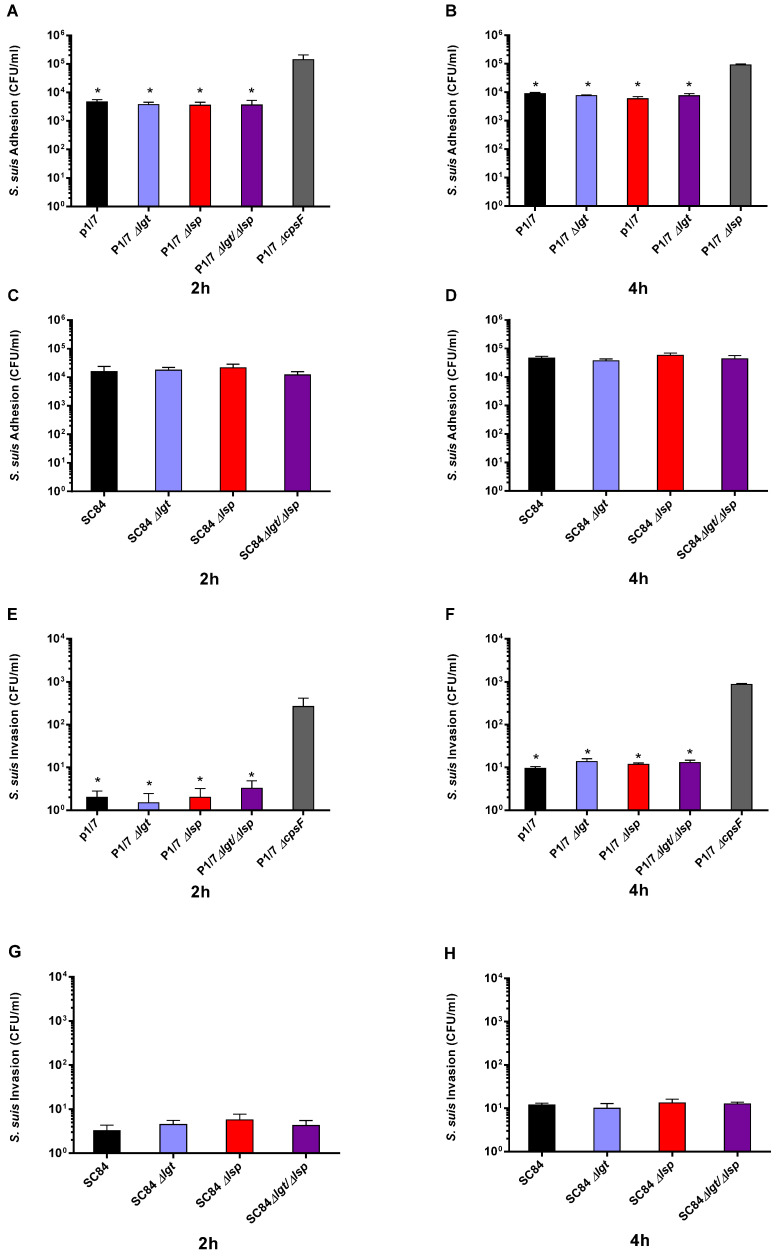
Adhesion (**A**–**D**) and invasion (**E**–**H**) of the *S. suis* serotype 2 wild-type virulent strain P1/7 (ST1) (**A**,**B**,**E**,**F**) and highly virulent strain SC84 (ST7) (C, D, G and H) (black) as well as their respective Δ*lgt* (blue), Δ*lsp* (red) and Δ*lgt*/Δ*lsp* (purple) mutant strains to porcine tracheal epithelial cells after 2 h (**A**,**C**,**E**,**G**) or 4 h (**B**,**D**,**F**,**H**) of incubation. * (*p* < 0.05) indicates a significant difference between the Δ*cpsF* mutant and wild-type strain or the Δ*lsp* and/or Δ*lgt*/Δ*lsp* mutant strains. Each bar represents the mean bacterial concentration (CFU/mL) + SEM (*n* = 3 independent experiments).

Due to their location on the bacterial surface, LPPs may interact with the extracellular environment and can facilitate bacterial adhesion to different substrates or host tissues [47]. However, the role of the lipoprotein maturation enzymes in streptococcal adhesion/invasion to cells is poorly known. Thus, the interactions between the two wild-type strains and their respective Δ*lgt*, Δ*lsp* and Δ*lgt*/Δ*lsp* mutants with porcine epithelial cells were studied herein. Wild-type serotype 2 strains and all mutants similarly adhered and (weakly) invaded porcine tracheal epithelial cells after 2 and 4 h of incubation, indicating no significant role of the Lsp and Lgt enzymes on these bacterial/cell interactions (Figure 2A–G). No differences were observed between the adhesion and invasion capacity of the wild-type strains. As expected, the non-encapsulated mutant (used as a positive control) significantly adhered to and invaded cells more than its wild-type strain (P1/7 ST1) [48] (Figure 2A,B,E,F).

### 3.3. Presence of the Prolipoprotein Signal Peptidase Type II Is Partially Required for S. suis Serotype 2 In Vitro Resistance to Bacterial Killing in a Whole Blood Test

**Figure 3:**
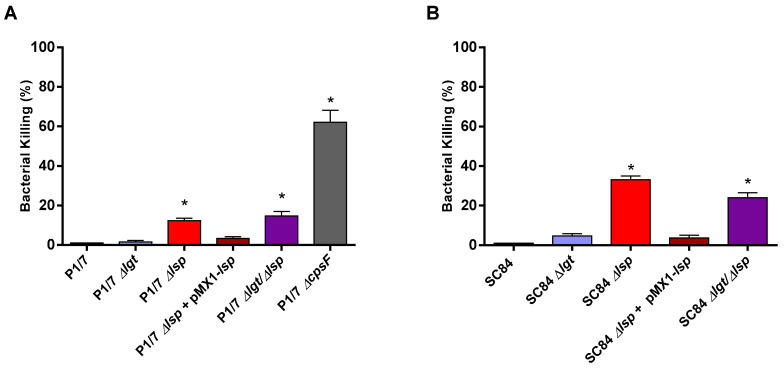
Capacity of the *S. suis* serotype 2 virulent wild-type ST1 strain P1/7 (**A**) and highly virulent ST7 strain SC84 (**B**) (black), and their respective Δ*lgt* (blue), Δ*lsp* (red), Δ*lsp* + pMX1-*lsp* (dark red) and Δ*lgt*/Δ*lsp* (purple) mutant strains to resist the bactericidal effect of murine whole blood after 2 h of incubation. Percentage of bacterial survival was calculated in comparison to bacteria in plasma alone. Data represent the mean + SEM (*n* = 3 independent experiments). * (*p* < 0.05) indicates a significant difference between the wild-type and mutant strains.

One of the most important steps in the pathogenesis of the *S. suis* infection is survival and dissemination in the bloodstream. *S. suis* must be able to survive in this hostile environment and to resist killing by neutrophils and monocytes and cause bacteremia followed by sepsis [7]. To evaluate the role of LPP maturation on bacterial killing resistance, a bactericidal test with mouse whole-blood was used. The non-encapsulated serotype 2 Δ*cpsF* mutant (derived from the wild-type P1/7 strain and used as a positive control) was killed at high rates when compared to the wild-type strain, confirming previous data [48] (Figure 3A). As expected, both wild-type strains were similarly highly resistant to bacterial killing (less than 3% of killing). Both Δ*lsp* mutants were killed at significantly higher rates (between 15% and 25%) than their respective wild-type strains, while the complemented strains presented low bacterial killing rates, similar to those of their respective wild-type strains. However, both Δ*lgt* mutants presented low killing rates similar to those of their respective wild-type strains. The reduced resistance to bacterial killing of the Δ*lgt*/Δ*lsp* double mutants was similar to that observed for the Δ*lsp* mutants for both strains (Figure 3A,B), indicating no additive effect.

### 3.4. The Diacyl Motif and the Peptide Signal Cleavage Are Important for the Recognition of Periplasmic and/or Secreted S. suis Serotype 2 Lipoproteins by Innate Immune Cells

**Figure 4:**
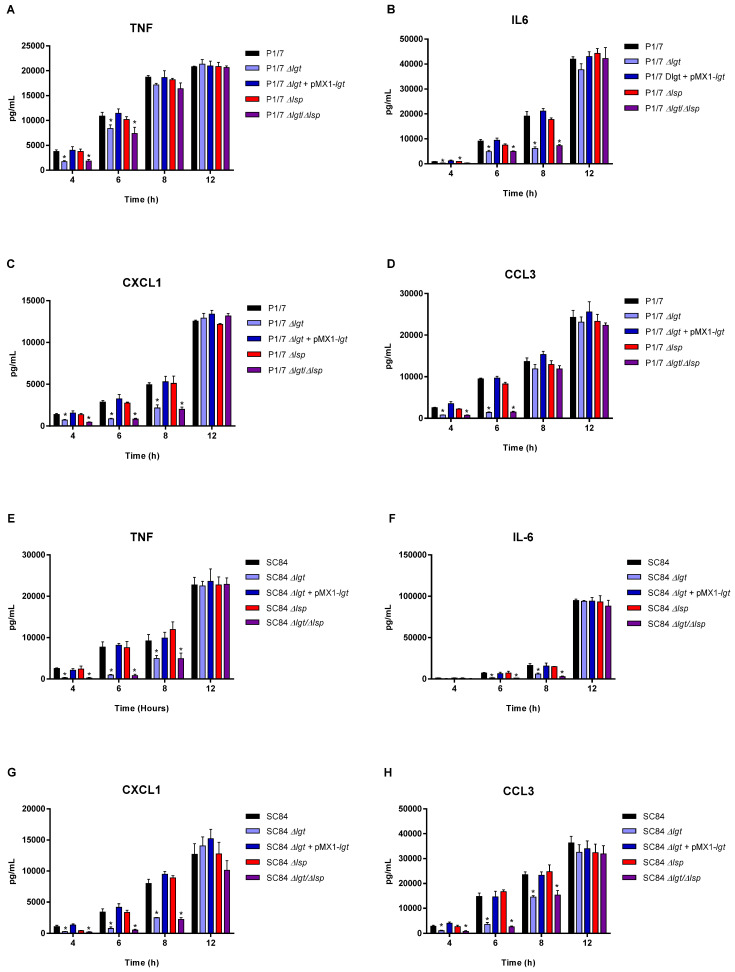
Pro-inflammatory mediator production by bmDCs following infection with live bacteria of the *S. suis* serotype 2 virulent wild-type ST1 strain P1/7 (**A**–**D**) and highly virulent ST7 strain SC84 (**E**–**H**) (black), as well as their respective Δ*lgt* (blue), Δ*lsp* (red) and Δ*lgt*/Δ*lsp* (purple) mutant strains and the Δ*lgt* + pMX1-*lgt* complemented strain (dark blue). Production of TNF (**A**,**E**), IL-6 (B and F), CXCL1 (**C**,**G**) and CCL3 (**D**,**H**). Data represent the mean + SEM (*n* = 4 independent experiments). * (*p* < 0.05) indicates a significant difference between the wild-type and mutant strains. Mock-infected cells induced negligible cytokine values < 300 pg/mL (not shown).

**Figure 5:**
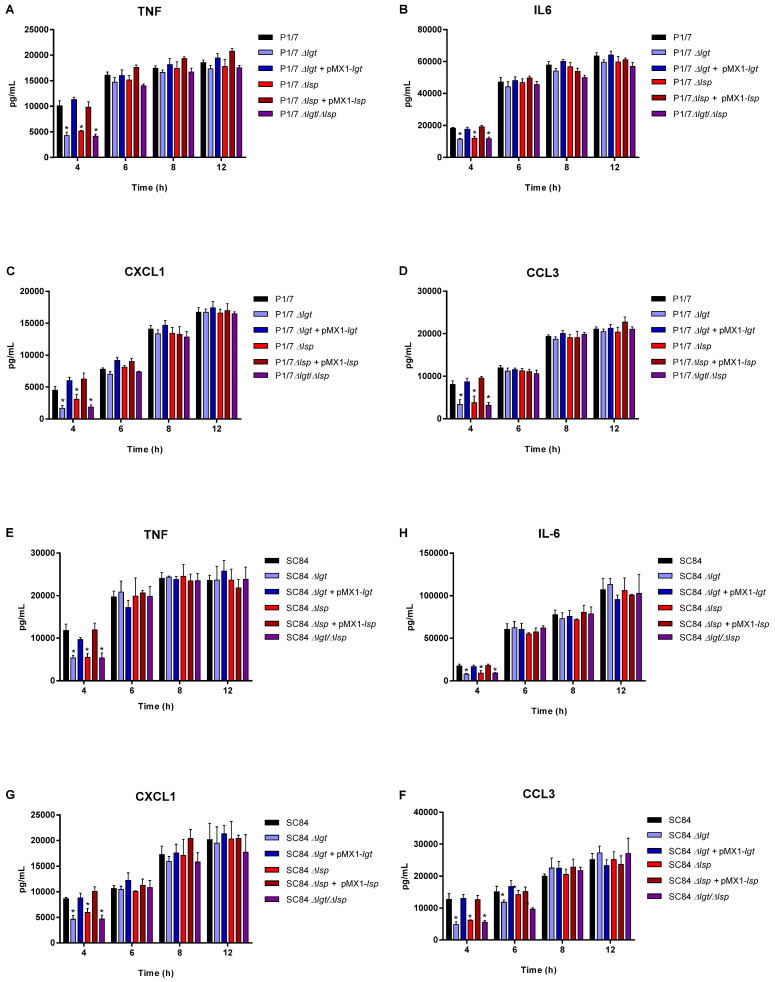
Pro-inflammatory mediator production by bmDCs following infection with heat-killed bacteria of *S. suis* serotype 2 virulent wild-type ST1 strain P1/7 ST1 (**A**–**D**) and highly virulent ST7 strain SC84 (**E**–**H**) (black), as well as their respective Δ*lgt* (blue), Δ*lsp* (red) and Δ*lgt*/Δ*lsp* (purple) mutant strains and the Δ*lgt* + pMX1-*lgt* (dark blue) or Δ*lsp* + pMX1-*lgt* complemented strains (dark red). Production of TNF (**A**,**E**), IL-6 (**B** and **F**), CXCL1 (**C**,**G**) and CCL3 (**D** and **H**). Data represent the mean + SEM (*n* = 4 independent experiments). * (*p* < 0.05) indicates a significant difference between the wild-type and mutant strains. Mock-infected cells induced negligible cytokine values < 300 pg/mL (not shown).

**Figure 6:**
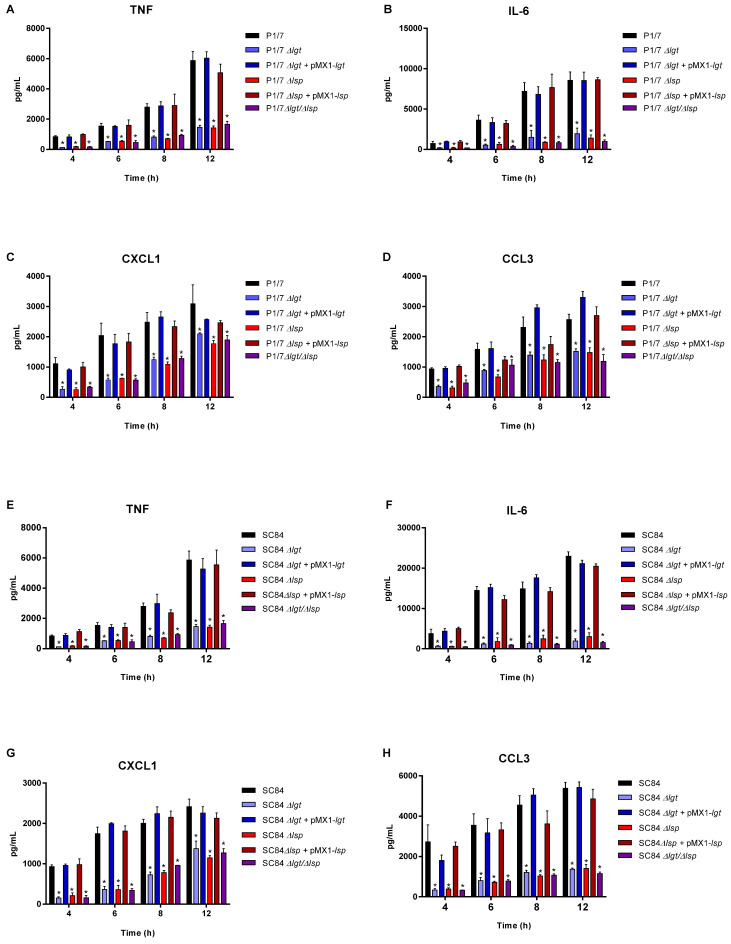
Pro-inflammatory mediator production by bmDCs following infection with bacterial-free supernatant of *S. suis* serotype 2 virulent wild-type ST1 strain P1/7 (**A**–**D**) and highly virulent ST7 strain SC84 (**E**–**H**) (black), as well as their respective Δ*lgt* (blue), Δ*lsp* (red) and Δ*lgt*/Δ*lsp* (purple) mutant strains and the Δ*lgt* + pMX1-*lgt* (dark blue) or Δ*lsp* + pMX1-*lgt* complemented strains (dark red). Production of TNF (**A**,**E**), IL-6 (**B**,**F**), CXCL1 (**C**,**G**) and CCL3 (**D**,**H**). Data represent the mean + SEM (*n* = 4 independent experiments). * (*p* < 0.05) indicates a significant difference between wild-type and mutant strains. Mock-infected cells induced negligible cytokine values < 300 pg/mL (not shown).

bmDCs were used as an innate immune cell model given that DCs play a critical role during *S. suis* pathogenesis and that their inflammatory response to *S. suis* serotype 2 has been well-characterized [10,27]. bmDCs were activated for up to 12 h with live bacteria for evaluation of the role of periplasmic and secreted LPPs (Figure 4), with heat-killed bacteria for the evaluation of periplasmic LPPs (Figure 5) or with bacterial-free supernatant to study the activation potential of secreted LPPs (Figure 6).

For all experiments and at all incubation times, control mock infected cells presented negligible cytokine values < 300 pg/mL (not shown). For both strains, the live Δ*lgt* and double Δ*lgt*/Δ*lsp* mutants (but not the Δ*lsp* mutants) induced lower levels of the different pro-inflammatory mediators during early incubation times (between 4 and 8 h after bacterial/cell contact), although no differences were observed at 12 h of incubation for all mediators (Figure 4). This early effect was more evident when using washed heat-killed bacteria, where differences were only observed at 4 h of incubation (Figure 5). The MOI used for killed bacteria was 100 times higher than that used at the beginning of the incubation time with live bacteria. However, both the diacyl motif and the peptide signal cleavage were shown to be similarly important for the recognition of secreted lipoproteins even after 12 h of incubation (Figure 6). The deletion of both genes was not additive, since all values observed with the double Δ*lgt*/Δ*lsp* mutant were always similar to the individual mutants. In all cases, complemented mutants behaved similar to wild-type strains, confirming the influence of the gene deletion (Figure 4, Figure 5 and Figure 6).

### 3.5. Absence of the Lgt and/or Lsp Enzymes Affects S. suis Serotype 2 Virulence in a Strain-Dependent Manner

**Figure 7:**
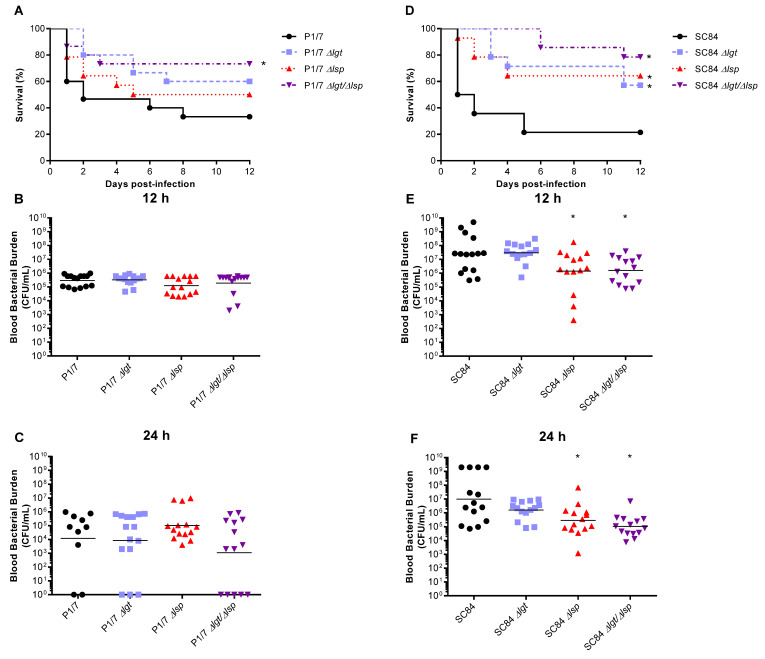
Survival (**A**,**D**) and blood bacterial burden at 12 h (**B**,**E**) and 24 h post-infection (**C**,**F**) of C57BL/6 mice following intraperitoneal inoculation of the *S. suis* serotype 2 virulent wild-type ST1 strain P1/7 and highly virulent ST7 strain SC84 (black) as well as their respective Δ*lgt* (blue), Δ*lsp* (red) and Δ*lgt*/Δ*lsp* (purple) mutant strains. Data represent survival curves (**A**,**D**) (*n* = 15) or geometric mean (**B**,**C**,**E**,**F**) (n = survived mice at each time point). * (*p* < 0.05) indicates a significant difference between survival or blood bacterial burden of mice infected mutant strains when compared to their respective wild-type strain.

To evaluate the role of the LPP maturation enzymes in the systemic virulence of both *S. suis* serotype 2 strains, a well-characterized C57BL/6 mouse model of infection was used [29,31]. As expected, the wild-type ST7 SC84 strain presented a higher level of virulence than the ST1 strain P1/7 [26]. For the ST1 strain P1/7, the absence of the Lgt or Lsp enzymes did not significantly affect strain virulence. Only the Δ*lgt*/Δ*lsp* double mutant was significantly less virulent than the wild-type strain (*p* < 0.05) (Figure 7A). Conversely, for the ST7 strain SC84, both single mutants as well as the Δ*lgt*/Δ*lsp* double mutant were significantly less virulent than the wild-type strain (*p* < 0.05) (Figure 7D). No significant differences between single and double mutant were observed for both strains.

Blood bacterial burden was evaluated at the early infection times of 12 h (Figure 7B,E), 24 h (Figure 7C,F) and 48 h (not shown) p.i. For the ST1 strain P1/7, no differences were observed between the acute blood bacterial burden of mice infected with the wild-type or mutants (Figure 6B,C). Conversely, for the ST7 strain SC84, although no significant differences were observed with the Δ*lgt* mutant, a slight decrease in blood bacterial burden was observed with the Δ*lsp* mutant at 12 and 24 h, but not at 48 h p.i., which was also observed with the Δ*lgt*/Δ*lsp* double mutant strain (Figure 6E,F) (not shown). However, it is difficult to ascertain if the differences observed (although statistically significant) had a real biological impact.

### 3.6. Absence of the Lsp and/or Lgt Enzymes Reduces the In Vivo Inflammatory Response after Infection of Mice with Both Wild-Type S. suis Serotype 2 Strains

**Figure 8:**
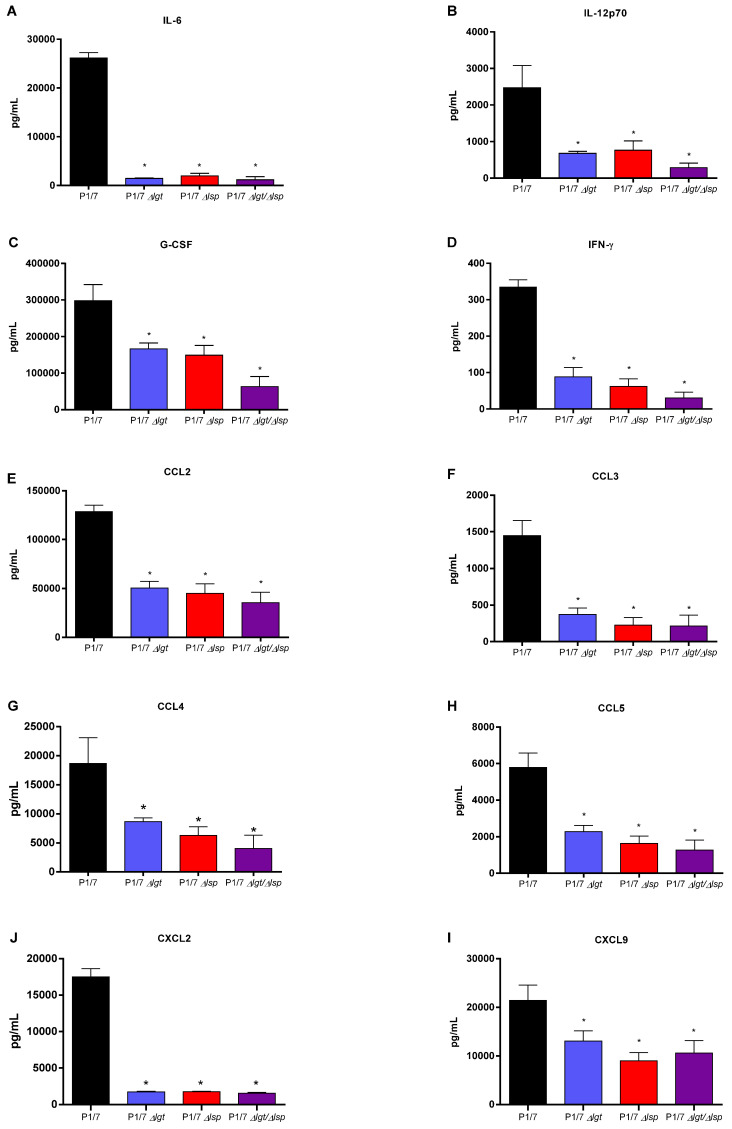
Plasma levels of IL-6 (**A**), IL-12p70 (**B**), G-CSF (**C**), IFN-γ (**D**), CCL2 (**E**), CCL3 (**F**), CCL4 (**G**), CCL5 (**H**), CXCL9 (**I**) and CXCL2 (**J**) in mice 12 h following intraperitoneal inoculation of the *S. suis* serotype 2 virulent wild-type ST1 strain P1/7 (black) or Δ*lgt* (blue), Δ*lsp* (red) and Δ*lgt*/Δ*lsp* (purple) mutant strains. Data represent mean + SEM (*n* = 8 individuals). * *p* < 0.05 indicates a significant difference between plasma levels of mice infected with the mutant strains when compared to the wild-type strain.

**Figure 9:**
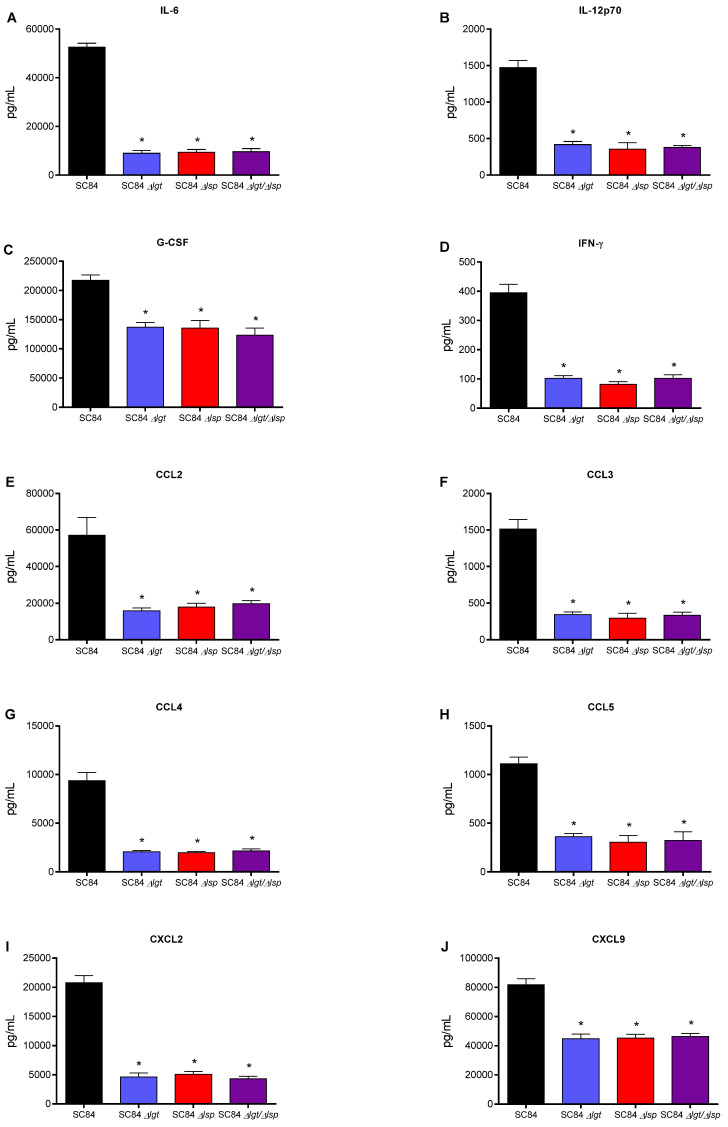
Plasma levels of IL-6 (**A**), IL-12p70 (**B**), G-CSF (**C**), IFN-γ (**D**), CCL2 (**E**), CCL3 (**F**), CCL4 (**G**), CCL5 (**H**), CXCL9 (**I**) and CXCL2 (**J**) in mice 12 h following intraperitoneal inoculation of the *S. suis* serotype 2 highly virulent wild-type ST7 strain SC84 (black) or Δ*lgt* (blue), Δ*lsp* (red) and Δ*lgt*/Δ*lsp* (purple) mutant strains. Data represent mean + SEM (*n* = 8 individuals). * *p* < 0.05 indicates a significant difference between plasma levels of mice infected with the mutant strains when compared to the wild-type strain.

To evaluate the inflammatory response of animals infected with the different wild-type and mutant strains, plasma mediators were evaluated after 12 h of infection. Using the single and double Δ*lgt*/Δ*lsp* mutant, similar results were observed for both the virulent and highly virulent strains. Concentrations of the different mediators tested (IL-6, IL-12p70, G-CSF, IFN-γ, CCL2, CCL3, CCL4, CCL5, CXCL2 and CXCL9) were significantly lower in mice infected with the different mutants when compared to their respective wild-type strain (Figure 8 and Figure 9). No additive effect was observed with the double Δ*lgt*/Δ*lsp* mutants when compared to the single mutants for both strains (*p* > 0.05). Consequently, the maturation of LPPs is implicated in early *S. suis* recognition by the immune system that may result in an exacerbated systemic inflammatory response.

## 4. Discussion

Although knowledge on the pathogenesis of the infection caused by *S. suis* has improved in recent years, the precise involvement of most putative virulence factors remains poorly known and further studies are necessary to elucidate this [5]. Virulent *S. suis* strains colonize the upper respiratory tract and from there, via still unknown mechanisms, invade the bloodstream, resist bacterial killing and disseminate, causing either sudden death (due to an excessive inflammatory reaction) or different pathologies depending on the targeted organs (mainly meningitis, arthritis or endocarditis) [49]. Many cell surface proteins have been suggested to play a role in virulence, mainly for serotype 2 (by far the most frequently studied serotype), such as those with a LPXTG-motif, those lacking a known C-terminal cell wall sorting signal, hemagglutinins, enzymes, different secreted factors as well as lipoproteins (LPPs) [50]. It has been estimated that LPPs account for approximately 2–3% of the pneumococcal proteome and about 40% of the indicated and predicted pneumococcal surface proteins [12]. However, few studies are available concerning the distribution and role of LPPs in *S. suis* strains [26]. To evaluate the role of LPPs maturation in the pathogenesis of the infection caused by this important swine and human pathogen, we characterized mutants defective for the Lgt and/or Lsp enzymes. Since the pathogenicity mechanisms used by virulent and highly virulent serotype 2 strains may vary [26,28], we included two different wild-type strains, ST1 and ST7, representing both groups of strains, respectively.

A large proportion of LPPs are substrate-binding proteins of ABC transporter systems responsible for the acquisition of multiple nutrients [16]. Growth in rich medium of the Δ*lgt* and Δ*lsp* mutants was not significantly affected for both the ST1 and ST7 strains. Two previous studies showed that inactivation of the *lgt* or *lsp* genes resulted in viable *S. suis* bacteria being able to efficiently grow (sometimes with a slightly increased lag phase [25]) in rich media [24]. In Gram-positive bacteria, the effect of those mutations on bacterial growth varies depending on the pathogen and the characteristic of the medium used (rich vs. poor medium). For example, restricted growth of a Δ*lgt* mutant of *Staphylococcus aureus* in media with limited nutrient has been reported [51]. Furthermore, absence of the Lgt enzyme in *S. pneumoniae* had no effect on bacterial growth in artificial media but resulted in a significant growth defect in blood and bronchoalveolar lavage fluids [18,21,22]. Likewise, Lsp deficiency caused an impaired intracellular growth of *Listeria monocytogenes*, a reduced growth of *Streptococcus mutants* in a minimal medium and a delayed growth of *S. pneumoniae* in rich medium [18]. We showed that growth of the Δ*lgt* and Δ*lsp S. suis* mutant strains also presented a normal growth in plasma, which is considered closer to in vivo conditions. Our results obtained with the double Δ*lgt*/Δ*lsp* mutants were similar to those observed with the individual Δ*lgt* or Δ*lsp* mutants, which indicates no additive effect when both enzymes are absent. Significant growth inhibition in rich medium was previously observed with a similar double mutant of *S. pneumoniae* [18].

The contribution of LPPs to streptococcal virulence has been linked to bacterial adhesion, invasion and immune evasion. Colonization is among the first steps of the *S. suis* pathogenesis, and it has been shown that LPPs may directly or indirectly participate in the early stages of other streptococcal infections, that is, adhesion and invasion of host cells. Examples of LPPs that have been described as adhesins/invasins are the laminin-binding proteins (Lmb or Lbp) of *Streptococcus agalactiae* [52] and *Streptococcus pyogenes* [53], respectively. A surface expressed laminin-binding protein (highly homologous to Lmb and Lbp) has also been described in *S. suis* [54], although its role as adhesin/invasin has not yet been studied. Studies on the role of Lsp and/or Lgt on the adhesion/invasion properties of pathogenic streptococci are scarce. Only one study reported that a *Streptococcus agalactiae* Δ*lgt* mutant adhered significantly less to endothelial cells than its corresponding wild-type strain [19]. Results from the current study indicate that *S. suis* mutants defective in LPP maturation are able to adhere to and invade porcine respiratory epithelial cells at similar rates when compared to their respective wild-type strains, suggesting that the LPP maturation enzymes are not essential for these bacterial-cell interactions. There are several possible explanations for these results. First, it is possible that LPPs do not play important roles in the *S. suis* interactions with host cells. Adhesion to laminin has been reported for a non-encapsulated *S. suis* mutant, unlike with its well-encapsulated wild-type strain, suggesting that some adhesins may be hindered by the presence of the CPS [55]. In addition, many other proteins (without the lipid moiety) have been shown to be implicated in adhesion/invasion processes [50], which may indicate a certain redundancy. Another hypothesis includes the possibility that a lack of LPP maturation does not completely eliminate the functional activities of such proteins, as demonstrated for *Streptococcus equi* [23]. Finally, in the current study, we evaluated the interactions of *S. suis* with a single cellular type, and it has been hypothesized that the first steps of the infection are complex [49]. It has been reported that a *S. suis* Δ*lsp* mutant may colonize tonsils less efficiently than its wild-type strain after intranasal infection of germ-free piglets, demonstrating that further studies are needed to clarify the role of the LPP maturation enzymes in the first steps of the infection [24].

Once in the bloodstream, *S. suis* must survive and multiply for the development of disease. It has been demonstrated that the CPS is one of the main factors responsible for resistance to phagocytosis and killing [5]. However, different proteins (including LPPs) have also been reported as being implicated in *S. suis* survival in blood, as with other streptococci [56,57]. Our results showed that in the absence of the prolipoprotein signal peptidase type II (Lsp), *S. suis* seems to be somehow less resistant to bacterial killing using a whole-blood in vitro test. Similarly, bacteremia levels of mice infected with the Δ*lsp* and Δ*lsp*/Δ*lgt* mutants were slightly lower 12 and 24 h p.i. (but not at 48 h p.i.; not shown) for the ST7 strain SC84, but not for the ST1 strain P1/7. At this point, it is uncertain if these differences are biologically significant and further studies are required. The presence of the prolipoprotein diacylglyceryl transferase enzyme did not seem to significantly contribute to bacterial survival for any of the strains as shown in vitro and in vivo.

Although streptococcal LTA was originally thought to be responsible for cell activation, it was more recently demonstrated that co-purified LPPs are responsible for streptococcal activation, including for *S. suis* [11]. In general, results from the current study clearly indicated that lack of LPP maturation affects cell activation by both strains of *S. suis*. Secreted LPPs seem to be mainly affected since inflammatory induction by bacterial-free supernatant from all mutants was significantly reduced up to, at least, 12 h of incubation. Once again, the double Δ*lsp*/Δ*lgt* mutant did not present any additive inhibition and the impact of both enzymes seems to be redundant. When the bacterial supernatant was removed (heat-killed bacteria), the inhibitory effect of all mutants was similar at early incubation times (4 h) only, whereas only the absence of the diacyl motif influenced cell activation when live bacteria was used. Periplasmic and secreted LPPs seem to be differently affected in the absence of maturation enzymes. It has been reported that in absence of Lsp, some of the LPPs of *S. pneumoniae* are found in reduced quantities in the extracellular environment [18]. This would be due to the additional presence of the hydrophobic peptide signal, which further binds lipoproteins at the membrane level. The peptide signal is known to contribute to the transient attachment of pre-prolipoproteins once secreted outside the cell before they undergo the various stages of maturation [18]. More studies are needed to study the localization of *S. suis* LPPs in those mutants. Other surface or secreted proteins not regulated by the Lsp and Lgt enzymes are also cell activators [58,59].

The role of the LPP maturation enzymes in virulence has been studied in other Gram-positive bacteria through the characterization of Lgt or Lsp mutants with variable results [14,22,23]. The Lgt enzyme contributes to virulence of *S. pneumoniae* [21] and *S. aureus* [51] in mouse infection models. The Lsp enzyme is required for the full virulence of *L. monocytogenes* [60], *Mycobacterium tuberculosis* [13] and *S. aureus* [61]. In the current study, and using a mouse model of systemic infection, it was shown that the Lgt and Lsp enzymes influence the virulence of the highly virulent *S. suis* serotype 2 ST7 strain SC84. All three Δ*lgt*, Δ*lsp* and Δ*lgt*/Δ*lsp* mutants induced similar and significantly less mortality than the wild-type strain. As mentioned above, the slightly lower bacterial burdens for the Δ*lsp* and Δ*lgt*/Δ*lsp* mutant strains for the highly virulent strain at early times p.i. would probably not explain by itself the difference in virulence. For the virulent ST1 strain P1/7, the Δ*lgt* or Δ*lsp* mutants were not significantly less virulent when compared to the wild-type strain. Reasons for these differences are not completely understood, although it has been previously shown that ST1 and ST7 strains may use different pathogenic mechanisms [26]. Only the double Δ*lgt*/Δ*lsp* mutant was significantly less virulent when compared to its wild-type strain. There is only one previous study addressing the virulence of such a double-mutant, with Group B *Streptococcus* in an atypical drosophila model, showing a significant synergic decreased virulence potential of this mutant [62]. In the case of *S. suis*, there is only one study showing that a Δ*lsp* mutant, also derived from a serotype 2 ST1 strain, was as virulent as its wild-type strain in an intranasal piglet competition infection model, using germ-free animals [24].

Finally, another possible explanation for the observed differences in virulence with the highly virulent ST7 SC84 mutants is a potentially lower induction of the inflammatory response, which is generally associated with *S. suis* clinical signs/mortality. LPPs are the main activators of TLR2 in streptococci, including *S. suis* [11]. At early p.i. times (12 h), levels of all inflammatory mediators tested were significantly lower for all mutants when compared to their respective wild-type strains. It has been shown that regulation of protective/detrimental inflammatory mediators clearly differ between these two strains [29], and a late activation of pro-inflammatory cytokines and chemokines by LPPs of the highly virulent wild-type ST7 strain cannot be ruled out. A final confirmation of virulence should be evaluated using a systemic model of infection in conventional pigs, being the natural host. However, these models are complicated due to high variation in animal susceptibility [5]. In addition, even if an intraperitoneal model has been developed for use in pigs (similar to that used in mice in the current study), a high concentration of bacteria must be injected to animals in order to observe clinical signs [63]. Subtle differences in virulence (as those observed in mice with the Δ*lgt* and/or Δ*lsp* mutants) would be highly difficult to observe in such models.

## 5. Conclusions

In summary, our study suggests that LPP maturation does not generally seem to play an important role in adhesion to and invasion of porcine epithelial cells. However, they regulate dendritic cell activation in vitro and host activation at early time points after infection. In addition, these enzymes seem to play a differential role in virulence depending on the genetic background of the strain (ST1 vs. ST7). Since it has been reported that the CPS only partially masks sub-capsular domains and bacterial wall components of North-American *S. suis* serotype 2 ST25 strains, these would be more exposed [40]. As such, a greater role of the enzymes responsible for LPP maturation would be expected in these strains, and future studies are warranted.

## Figures and Tables

**Table 1:** List of strains and plasmids used in this study.

Strain or plasmid	Characteristics	Reference
*Streptococcus suis*		
P1/7	Virulent serotype 2 ST1 strain isolated from a case of pig meningitis in the United Kingdom	[32]
P1/7Δ*lgt*	Isogenic mutant derived from P1/7; in frame deletion of the *lgt* gene	This study
P1/7Δ*lsp*	Isogenic mutant derived from P1/7; in frame deletion of the *lsp* gene	This study
P1/7Δ*lgt*/Δ*lsp*	Isogenic mutant derived from P1/7; in frame deletion of the *lgt* and *lsp* genes	This study
P1/7 comp Δ*lgt*	Mutant Δ*lgt* complemented with the pMX1-*lgt* complementation vector	This study
P1/7 comp Δ*lsp*	Mutant Δ*lsp* complemented with the pMX1-*lsp* complementation vector	This study
SC84	High virulent serotype 2 ST7 strain isolated from a human streptococcal toxic shock-like syndrome case in China	[33]
SC84Δ*lgt*	Isogenic mutant derived from SC84; in frame deletion of the *lgt* gene	This study
SC84Δ*lsp*	Isogenic mutant derived from SC84; in frame deletion of the *lsp* gene	This study
SC84Δ*lgt*/Δ*lsp*	Isogenic mutant derived from SC84; in frame deletion of the *lgt* and *lsp* genes	This study
SC84 comp Δ*lgt*	Mutant Δ*lgt* complemented with the pMX1-*lgt* complementation vector	This study
SC84 comp Δ*lsp*	Mutant Δ*lsp* complemented with the pMX1-*lsp* complementation vector	This study
*Escherichia coli*		
TOP10	F^−^ *mrcA* Δ(*mrr-hsd*RMS-*mcr*BC) φ80 *lacZ*ΔM15 Δ*lac*X74 *rec*A1 *ara*D139 Δ(*araleu*) 7697 *gal*U *gal*K *rps*L (Str^R^) *end*A1 *nup*G	Invitrogen
MC1061	Host for pMX1 derivatives	[34]
**Plasmids**		
pCR2.1	Ap^r^, Km^r^, pUC *ori*, *lac*ZΔM15	Invitrogen
pSET4s	Spc^r^, pUC *ori*, thermosensitive pG+host3 *ori*, *lac*ZΔM15	[35]
pMX1	Replication functions of pSSU1, MCS pUC19 *lac*Z SpR, *mal*X promoter of *S. suis*, derivative of pSET2	[35,36]
p4Δ*lg* p4Δ*lsp*	pSET-4s carrying the construct for *lgt* allelic replacement pSET-4s carrying the construct for lsp allelic replacement	This study This study
pMX1-*lgt*	pMX1 carrying the intact *lgt* gene	This study
pMX1-*lsp*	pMX1 carrying the intact *lsp* gene	This study

**Table 2:** List of oligonucleotide primers used in this study.

Name	Sequence (5′–3′)	Construct
*lgt*-ID1	GGAACGCTATGGAACAGGTC	p4Δ*lgt*
*lgt*-ID2	CACTCCATGAAAAGGCGACG	p4Δ*lgt*
*lgt*-ID3	CGTAGACGGCCAAAATTCC	p4Δ*lgt*
*lgt*-ID4	CGCTTATCTGCTGGATTCTCC	p4Δ*lgt*
*lgt*-ID5	GCCAATCGTCTGCATCAAGG	p4Δ*lgt*
*lgt*-ID6	GGGTTGATAGAATGGGATTGCATACCAACG	p4Δ*lgt*
*lgt*-ID7	CGTTGGTATGCAATCCCATTCTATCAACCC	p4Δ*lgt*
*lgt*-ID8	GACCGACTTGCTGGTCAAAC	p4Δ*lgt*
*lsp*-ID1	TGAGAAAACTGTTGTGGGTA	p4Δ*lsp*
*lsp*-ID2	AGAGCACCAGCAATCATCAA	p4Δ*lsp*
*lsp*-ID3	TTGATGATTGCTGGTGCTCT	p4Δ*lsp*
*lsp*-ID4	TAGACAGCGAACAGAGATAC	p4Δ*lsp*
*lsp*-ID5	GCGCCTGCAGGATGATTTGGCGAACAGAAA	p4Δ*lsp*
*lsp*-ID6	ACCTACACCAACTGTTAATACTACCATCAA	p4Δ*lsp*
*lsp*-ID7	TTGATGGTAGTATTAACAGTTGGTGTAGGT	p4Δ*lsp*
*lsp*-ID8	CGCGCTGCAGTTTTAGTGTTTTCCTTGGGC	p4Δ*lsp*
pMX1-*lgt*-F	CCGCCATGGACAGATGGGGTTTGATGCAAC	pMX1-*lgt*
pMX1-*lgt*-R	CGCGAATTCGGACAAGGCAATAATCAAGAC	pMX1-*lgt*
pMX1-*lsp*-F	GTGCCATGGACTTTATTGAAACCATGCAGG	pMX1-*lsp*
pMX1-*lsp*-R	ATCGAATTCAATACCACCAACCTCAACTCT	pMX1-*lsp*

## Data Availability

Not applicable.
